# Parental rejection and generalized anxiety disorder in early adolescents: the mediating role of alexithymia

**DOI:** 10.3389/fpsyg.2025.1511983

**Published:** 2025-05-30

**Authors:** Matteo Angelo Fabris, Claudio Longobardi, Elżbieta Zdankiewicz-Ścigala, Dawid Konrad Ścigała

**Affiliations:** ^1^Department of Psychology, University of Turin, Turin, Italy; ^2^Faculty of Psychology, SWPS University, Warsaw, Poland; ^3^Institute of Psychology, The Maria Grzegorzewska University, Warsaw, Poland

**Keywords:** paternal rejection, generalized anxiety disorder, alexithymia, early adolescence, maternal rejection

## Abstract

The aim of this study is to extend our knowledge of the possible association between (maternal and paternal) parental rejection and generalized anxiety disorder (GAD) symptoms in early adolescence by investigating the possible mediating role of alexithymia. A sample of Italian early adolescents (*N* = 234; Mage: 11.86, *SD* = 0.93; 47% male) was recruited from several schools in northwestern Italy. Participants completed an anonymous self-report questionnaire that included information on experience of parental rejection, alexithymia, and GAD symptoms. Our results show an association between parental rejection (both maternal and paternal) and GAD symptoms. Furthermore, the data show that alexithymia tends to mediate the relationship between maternal/paternal rejection and GAD. In this sense, parental rejection is associated with an increase in alexithymic symptoms, which in turn is associated with an increase in GAD symptoms in early adolescents. Limitations of the research and practical implications are discussed.

## Introduction

Children and adolescents are a particularly high-risk population for the development of anxiety symptoms (Lin et al., 2024; Rapee et al., 2023), although the age of onset and development of the different forms of anxiety disorders (e.g., specific phobia, panic disorder, separation anxiety, etc.) tends to vary widely (Ohannessian et al., 2017; Rapee et al., 2023). Generalized anxiety disorder (GAD) is one of the most common anxiety disorders in adolescence and tends to occur in the transitional period between late childhood and early adolescence (Beesdo-Baum and Knappe, 2012; Ohannessian et al., 2017). GAD is a syndrome characterized by the experience of excessive, uncontrollable worry about various life circumstances (American Psychiatric Association, 2013). This disorder has a significant impact on the psychological and social functioning of adolescents and tends to become chronic and extend into adulthood (Crocetti et al., 2015).

Central to the worries of people with GAD are social-evaluative concerns in interpersonal interactions (Hudson and Rapee, 2004). This has led clinicians and researchers to focus on the close relationships of affected adolescents, and in particular the quality of their relationship with their parents, when investigating the onset of GAD symptoms. Indeed, despite the fact that during adolescence the individual experiences greater autonomy and the peer group becomes the main referent for emotional support, parents continue to exercise an important function in early adolescence and influence their psychological adjustment (Badenes-Ribera et al., 2019; Sechi et al., 2020). In this study, we will attempt to extend the current knowledge on the association between parental rejection (an emotionally abusive form of parenting experienced by children and characterized by a cold, neglectful, hostile or rejecting parental relationship) (Rohner, 2021) and the risk of developing GAD symptoms in a sample of early adolescents. In addition, our study will attempt to expand the current knowledge of the possible mechanisms involved in this relationship. Considering that several studies report that subjects with GAD tend to have difficulties in recognizing and regulating their emotions (Paniccia et al., 2018), and considering that this emotional competence has its roots in a close, warm and supportive relationship with parents, our study will investigate the possible role of alexithymia. Along these lines, we will seek to explore the possible mediating role of alexithymia in the relationship between both maternal and paternal parental rejection and GAD.

## Parental rejection and generalized anxiety disorder

Low quality of the relationship with parents appears to be a risk factor for the development of GAD symptoms in adolescence. In general, insecure attachment is considered a risk factor for anxiety disorders, and this association appears to be stronger in early adolescence (Colonnesi et al., 2011). One factor that negatively shapes the perception of the quality of parental relationships and promotes insecure attachment to the caregiver is parental rejection (Ildiz and Ayhan, 2020). According to the Interpersonal Acceptance-Rejection Theory (IPARTheory; Rohner et al., 2005), “parental warmth” is a key factor in promoting positive psychological adjustment in children, adolescents and adults. According to this theoretical perspective, “parental acceptance” is at one end of the warmth dimension, while “parental rejection” is at the other end (Rohner et al., 2005). Parental acceptance encompasses the warmth, affection, love and interest that parents show toward their children, while parental rejection describes the situation in which parents do not accept or want their children and perceive them as a burden (Rohner et al., 2005). Parental acceptance is expressed by parents with behaviors such as hugging, kissing, stroking, comforting and saying positive words. Parental rejection, which is conceptualized as a form of abuse, is expressed through behaviors such as coldness, deprivation of love, neglect, physical injury, humiliation, and criticism. According to theorists, parental rejection can be experienced by children as a combination of the following expressions: cold and unaffectionate, hostile and aggressive, indifferent and neglecting and undifferentiated rejecting (Khaleque and Rohner, 2012). Adolescents who have experienced rejection in the parental relationship tend to develop a more negative view of self-image and the social world and report a higher risk of developing psychological symptoms (Mendo-Lázaro et al., 2019; Khaleque and Rohner, 2002). Longitudinal evidence from different cultural contexts seems to indicate that both maternal and paternal rejection predict increased internalizing symptoms in adolescence (Rothenberg et al., 2022). In particular, some data suggest that parental rejection is significantly associated with increased GAD symptoms (Hale et al., 2006; Knappe et al., 2022). Overall, it is possible that a low quality of relationship with parents, characterized by a low level of affection and a high level of criticism and rejection, leads individuals to feel unsupported and unloved, establishing in them a negative self-perception and beliefs related to the risk of being rejected and abandoned by others (Badenes-Ribera et al., 2021; Colonnesi et al., 2011). In this way, the experience of parental rejection can be internalized by the individual, developing not only a negative self-image but also a negative expectation toward others, thinking that others will be prone to abandonment and rejection (Knappe et al., 2022), which fuels the anxious worries typical of GAD. This aspect is particularly evident in early adolescence, where the need for belonging and acceptance plays a key role in psychological adjustment processes (Fabris et al., 2024). However, one should be cautious in assuming a strict linear causal relationship between parental rejection and GAD, as some longitudinal studies suggest that GAD is a predictor of perceived parental rejection in adolescents (Hale et al., 2011; Nelemans et al., 2014). In this sense, it is possible that psychopathological symptoms influence self-perception in adolescents with GAD. For these adolescents, a negative self-evaluation could lead them to process social interactions in a way that is consistent with their self-worth, and therefore to perceive their relationship with their parents as more negative, critical and rejecting. Along these lines, adolescents could engage in behaviors that are consistent with their perceptions and thus stimulate more negative parental interactions, ultimately reinforcing perceptions of a loveless and negative relationship and thus higher perceived parental rejection (Nelemans et al., 2014).

In this study, we will examine the relationship between GAD symptoms and parental rejection by measuring paternal and maternal rejection separately. The reason for this is that the literature seems to indicate a differential role of maternal and paternal relationship quality in predicting childhood anxiety symptoms (Möller et al., 2016) and GAD in particular (van Eijck et al., 2012). Furthermore, research on parenting has primarily focused on the mother–child relationship, but we know that fathers can make a unique, significant contribution to child and adolescent development (Miranda et al., 2016). In addition, mothers and fathers may have different parenting attitudes and styles, and the literature seems to indicate that experienced parental dissimilarity in terms of emotional warmth or rejection has been linked to the development of internalizing problems (Miranda et al., 2016). Last but not least, although there is evidence for the association between low perceived parental relationship quality and GAD, few studies have specifically examined parental rejection in early adolescents. Furthermore, although theoretical and empirical evidence supports a possible link between GAD and parental rejection, research is still needed to understand what mechanisms might explain this link. In this vein, our study aims to test the possible mediating role of alexithymia in explaining the relationship between perceived maternal/paternal rejection and GAD symptoms in early adolescence.

### GAD and alexithymia

Alexithymia is a cognitive-affective disorder characterized by a diminished ability to identify and describe feelings, diminished imaginative capabilities, and a concrete and externally-oriented way of thinking (Taylor, 1984). Alexithymia is currently conceptualized as a condition characterized by a deficit in the subject’s awareness and verbal reporting of feelings, reflecting a disorder of emotion regulation (Paniccia et al., 2018; Scigala et al., 2024). Contemporary theorists define it as a multidimensional construct consisting of three interrelated (positively correlated) components: difficulty identifying feelings in oneself (DIF); difficulty describing feelings (DDF); and an externally orientated thinking style (EOT) in which one tends not to focus one’s attention on one’s feelings (Craparo et al., 2015).

In a study conducted among adolescents in Italy, a four-dimensional model of alexithymia was also tested. The fourth dimension is defined as the *lack of subjective significance or importance of emotions* (Craparo et al., 2015). The present research project employs an instrument that allows for the extraction of all four dimensions of alexithymia, each of which will be examined in the analyses.

The literature suggests that between 7 and 21 percent of adolescents exhibit alexithymic symptoms, with higher rates among younger adolescents and mixed results in terms of gender (Karukivi et al., 2010; Prino et al., 2019). Alexithymia appears to play a role in the development of internalizing disorders and anxiety syndromes in particular (Berardis et al., 2008; Karukivi et al., 2010; Prino et al., 2019), with alexithymic traits being quite common in individuals diagnosed with GAD (Wang et al., 2024). Studies from the psychiatric literature indicate that individuals with a GAD diagnosis tend to report higher levels of alexithymia (Onur et al., 2013; Wang et al., 2024) and that higher levels of alexithymia are associated with higher severity of GAD (Kumar et al., 2018). A recent study conducted with Italian adolescents (Paniccia et al., 2018) reported that 46 percent of adolescents with GAD had high levels of alexithymia, compared to 16 percent of the adolescent population without GAD. More specifically, compared to adolescents without GAD, adolescents with GAD reported higher scores on the sub-dimensions of alexithymia related to difficulties in describing feelings (DDF) and difficulties in identifying feelings (DIF), while no difference was found on the dimension of externally oriented thinking (EOT) (Paniccia et al., 2018). These data thus seem to reflect that GAD adolescents have difficulties identifying their feelings and understanding their emotional experience, and data seem to attribute a greater role to the affective components of alexithymia than to the cognitive component in the development of GAD in adolescence. Consistent with the “avoidance theory” of Borkovec et al. (2004), it is possible that people with GAD develop incessant worrying as a cognitive avoidance strategy to escape processing their feelings. Therefore, it is possible that adolescents with alexithymic symptoms, given their difficulties in recognizing and regulating their emotions, resort to worry as a cognitive strategy to avoid directly confronting the underlying emotions that they find too painful or disturbing. According to avoidance theory, worry then becomes a kind of “distraction” that prevents deep emotional processing but maintains a state of chronic anxiety. In addition, we should note that people with GAD often report marked functional somatic symptoms and increased somatosensory amplification as well as health-related anxiety (hypochondriasis) frequently presenting physical discomfort as their primary concern (Berardis et al., 2008). This could be due to the fact that people with alexithymia find it difficult to transfer emotions from the somatosensory to the representational level, so that they tend to express their psychological distress through physical symptoms (Berardis et al., 2008). Ultimately, then, the studies seem to indicate an association between alexithymia and GAD symptoms (Onur et al., 2013; Wang et al., 2024), suggesting at the basis of these symptoms a difficulty for sufferers to identify and describe their emotional states, and considering worries as an emotional suppression strategy in relation to emotional states that are perceived as painful or uncontrollable. However, despite early research suggesting a link between alexithymia and GAD symptoms in adolescence, no study has examined the potential mediating role of alexithymia in the relationship between parental rejection and GAD. This is a limitation given the importance of perceived parental relationship quality in predicting better emotional functioning in adolescents (Silvers, 2022).

### Alexithymia as a potential mediating factor between parental rejection and GAD

There is now ample evidence for the role that the relationship with parents in early childhood plays in shaping individuals’ emotional functioning (Cooke et al., 2019), and adolescents with alexithymic symptoms tend to report a more negative relationship with parents characterized by low levels of caring (Lukas et al., 2022; Thorberg et al., 2011). According to the multiple code theory of emotional information processing (Bucci, 2002), parents convey emotional information through facial expressions, gestures and spoken language. When a parent is emotionally available, the child feels supported in regulating their emotions through the acknowledgment of their feelings, the modeling of emotion regulation strategies, and the teaching of these strategies to the child. If the parent is insensitive, absent or rejecting for the child, the opportunities to learn adequate emotional knowledge from the parent are reduced, putting the individual at high risk of mismatching somatic sensations, emotional symbols and linguistic representations, which in turn promotes alexithymic symptoms. In this sense, if the parent is unavailable or rejecting, the child is exposed to increased distress and develops inappropriate strategies for emotion regulation, such as suppressing emotions. Parental rejection is seen as a form of emotional abuse in which the emotional needs for acceptance and closeness are completely ignored (Kosson et al., 2021). In such a relational context, children may have learned to suppress and internalize their distressing emotions rather than express and share them, yet internally focused feelings and complaints of somatic discomfort constituting high levels of alexithymia (Huang et al., 2022). Along these lines, a recent longitudinal study (van Strien et al., 2019) has shown that low quality of relationship with a caregiver in childhood is associated with more frequent use of emotional suppression, which in turn increases levels of alexithymia in adolescence. Research with adults, both in community populations (Hussain and Ahmed, 2014) and in clinical populations of individuals with substance dependence (Huang et al., 2022; Torrado et al., 2013), report that perceived parenting characterized by emotional unavailability and rejection tends to be associated with increased alexithymic symptoms (Huang et al., 2022; Torrado et al., 2013), and in particular with a reduction in the ability to describe and identify feelings (Torrado et al., 2013). In addition, adults with high levels of alexithymia tend to report negative mood regulation expectancies and fear of intimacy in relationships with romantic partners, probably in continuity with negative relationship experiences with attachment figures in childhood (Scigala et al., 2022, 2021).

Overall, the literature seems to indicate a link between parental rejection and alexithymia. In addition, some studies seem to indicate a possible mediating role between the perceived quality of the relationship with parents and the development of conditions closely associated with GAD. For example, in a Turkish study, the two sub-dimensions of difficulty identifying feelings (DIF) and difficulty describing feelings (DDF) were found to be significant in mediating the relationship between remembered maternal rejection and somatic complaints in adults (Işıl, 2023). However, literature about the relationships between parental rejection and GAD symptoms is very sparse, particularly in early adolescence, which is a limitation considering that this is a particularly critical developmental period and that both parental rejection and alexithymia may have important effects on adolescent psychological adjustment. Furthermore, no study has examined the possible mediating role of alexithymia in the relationship between parental rejection and GAD. This is a limitation considering that parental rejection appears to be associated with both alexithymia and GAD, and alexithymia may be a mechanism that can explain the association between GAD and previous experiences of parental rejection, adding to current knowledge about the maintenance and risk factors of GAD in adolescence.

#### Early adolescents as a target population

Our study focuses on early adolescents, not only because this developmental phase seems to be particularly susceptible to the occurrence of anxiety disorders and especially GAD symptoms, but also because major physical, psychological and social changes take place during this period, which often require parents to adapt their parenting practices (Seiffge-Krenke et al., 2010; Wang et al., 2011). During this developmental period, the parental relationship continues to play an important role in the formation of individual identity and provides emotional support and closeness (Badenes-Ribera et al., 2019). However, in early adolescence, individuals begin to take an increasing interest in peers as a source of support and intimacy, and in relation to the parental relationship, they begin to demand more autonomy, privacy and independence (Wang et al., 2011). Especially in early adolescence, they begin to renegotiate relationships with their parents (Wang et al., 2011). This can be a source of misunderstanding and conflict that can affect how adolescents perceive the quality of their parental relationship. Typically, during adolescence, closeness with parents decreases and negative interactions between children and their parents increase (Seiffge-Krenke et al., 2010), which may reinforce adolescents’ perceptions of a rejecting relationship. Nevertheless, parenting an adolescent is a challenging experience whose success depends largely on the parents’ ability to respect their child’s autonomy, and not all parents are able to provide warmth and support or deal appropriately with negativity (Seiffge-Krenke et al., 2010; Steinberg, 2001). Therefore, these changes may be perceived by parents as a source of worry and stress, which could affect the quality of the parent–child relationship. There is also evidence that rejecting, neglectful and less warming parenting is more strongly associated with internalizing symptoms in early adolescence than in childhood (Pinquart, 2017).

### The aim of the study

Based on the previous arguments, the aim of our study is to investigate the possible association between parental rejection and generalized anxiety disorder in early adolescents by investigating the possible mediating role of alexithymia.

Specifically, we expect that both maternal and paternal rejection are positively associated with GAD symptoms. Furthermore, we hypothesize that maternal and parental rejection are associated with increased alexithymic symptoms, which, in turn, are associated with increased GAD symptoms.

Focusing on the mediating role of alexithymia, it was decided to examine not only the mediating effect of the overall alexithymia score but also to identify which specific subdimension would serve as the strongest mediator. Accordingly, we hypothesize that difficulty identifying feelings (DIF) and difficulty describing feelings (DDF) will function as stronger mediators than externally oriented thinking (EOT) and lack of subjective significance or importance of emotions (LSS).

## Method

### Procedure and participants

The study involved 234 Italian adolescents aged 10–14 years (*M* = 11.86, SD = 0.93). The sample included 124 girls and 110 boys. Among the participants, 81% lived with both parents, and the majority had either one sibling (53.4%) or two siblings (21.8%). The sample was drawn from a community population of Italian adolescents recruited through three middle schools located in northwestern Italy (Piedmont and Lombardy regions). The schools were contacted by the principal investigator, who explained the purpose and procedures of the study to the school principals. Once approval was obtained from the school, the parents of the students were given permission for their child to take part in the study. Only those students whose parents had given their consent were included in the study. Further inclusion criteria were: sufficient reading and writing skills to participate in the study and native Italian speakers or comparable language skills. Adolescents were informed about the purpose and methods of the study, in particular about the protection of their anonymity. After the presentation, the students were free to participate in the study or not. No rewards were offered for participating in the study. The study complies with the guidelines of the Helsinki Convention and the Italian Association of Psychology (AIP). In addition, the study was approved by the Ethics Committee of the University of Turin.

### Measures

**Socio-demographic variables**. Participants completed an anonymous questionnaire containing a section of socio-demographic data that included: age, gender, living with parents; number of siblings, grade of school level.

**Parental rejection**. Perceived Parental Rejection was measured using the Italian version of the Parental Acceptance and Rejection Questionnaire-child version (PARQ; Rohner and Khaleque, 2005). The PARQ is a self-report questionnaire consisting of 24 items to be answered by the subject on a Likert scale ranging from 1 (*Never*) to 4 (*Every day*). The adolescents completed both the maternal and paternal versions of the PARQ, which assess maternal and paternal rejection, respectively. High scores on this measure indicate high experience of parental acceptance and low experience of parental rejection. The reliability of the scale for the overall score of maternal acceptance/rejection, assessed using Cronbach’s alpha *α* = 0.84 and McDonald’s omega *ω* = 0.84, was calculated based on the current dataset. Similarly, the reliability of the scale for the overall score of paternal acceptance/rejection, assessed using Cronbach’s alpha *α* = 0.89 and McDonald’s omega *ω* = 0.89, was also computed using the data from the present study.

**Generalized Anxiety Disorder**. Generalized Anxiety Disorder (GAD) was measured using The Generalized Anxiety Disorder-7 item (GAD-7) in its validated Italian version (Spitzer et al., 2006). GAD-7 is a brief, 7-item self-report tool evaluating symptoms of GAD. Each item is rated based on frequency using a 4-point scale ranging from “not at all” to “nearly every day,” focusing on the last 2 weeks. GAD-7 total scores range from 0 to 21. The reliability of the scale for the overall score, assessed using Cronbach’s alpha *α* = 0.84 and McDonald’s omega *ω* = 0.84, was calculated based on the current dataset.

**Alexithymia**. The Italian version of the Toronto Alexithymia Scale-20 (Craparo et al., 2015) was used to assess alexithymia. The TAS-20 is a self-report measure of alexithymia. We used a version of the instrument that was specifically validated in the Italian population of early adolescents. TAS-20 consists of four subscales: Difficulty identifying feeling (DIF) (7 items); Difficulty describing feelings (DDF) (4 items); Externally oriented-thinking (EOT) (4 items); and Lack of subjective significance or importance of emotions (LSS) (4 items). The total alexithymia score is the sum of all items, whilst the score for each subscale factor is the sum of specific items.

The reliability of the total alexithymia score was satisfactory, with Cronbach’s *α* = 0.77 and McDonald’s omega *ω* = 0.79. Regarding the individual subscales: Difficulty Identifying Feelings (DIF): *α* = 0.89, *ω* = 0.89; Difficulty Describing Feelings (DDF): *α* = 0.70, *ω* = 0.69; Externally Oriented Thinking (EOT): *α* = 0.52, *ω* = 0.50; Lack of Subjective Significance (LSS): *α* = 0.51, *ω* = 0.50.

### Data analysis

At the beginning, the analysis of the data was focused on assessing the distribution of variables and searching for outliers using measures of central tendency and dispersion as well as tests of the normality of the distribution. Assessment of normality followed the recommendations of Byrne (2010) and Tabachnick and Fidell (2013). In addition to the Shapiro–Wilk test, skewness and kurtosis values were examined for each variable. Values falling within the range of ±2 were considered acceptable for the assumption of normality. Visual inspections of histograms and Q–Q plots were also conducted. Descriptive statistics and a correlation matrix of variables were then analyzed using Pearson’s product–moment correlation coefficient.

The principal aim of the results presentation was to investigate the mediating function of alexithymia and its subdimensions in the relationship between maternal and paternal rejection and the manifestation of generalized anxiety disorder (GAD) symptoms. Initially, a model was tested in which the overall level of alexithymia was examined as a mediator. Subsequently, the analysis focused on identifying which specific dimension of alexithymia served as the strongest mediator in the relationship between parental rejection and symptoms of Generalized Anxiety Disorder (GAD). This analysis was conducted using a non-standard macro for SPSS Process (Hayes, 2013), specifically Model 4, which allows for the examination of mediation effects.

In order to better confirm the validity of the mediation model, it was decided to use the bootstrapping method with a sampling of 10,000 and thus the analysis of confidence intervals with 95% probability.

## Results

To substantiate the subsequent results derived from theoretical assumptions, the initial analysis focuses on examining the relationship between parental rejection (from both mother and father) and alexithymia, as presented in Table 1.

**Table 1:** Descriptive statistics and correlation matrix concerning the relationships between parental rejection, alexithymia, and generalized anxiety disorder.

	*M*	SD	MIN	MAX	2 GAD	3 PARQ Mother	4 PARQ Father	5 TAS	6 DIF	7 DDF	8 EOT	9 LSS
1 Age	11.86	0.93	10	14	0.12	−0.08	−0.06	0.10	0.09	0.142*	0.02	0.01
2 GAD	6.20	5.00	0	21		−0.336**	−0.268**	0.656**	0.676**	0.524**	0.04	0.377**
3 PARQ Mother	58.79	6.95	33	73			0.386**	−0.347**	−0.324**	−0.293**	−0.12	−0.154*
4 PARQ Father	57.52	9.41	21	73				−0.308**	−0.275**	−0.241**	−0.12	−0.171*
5 TAS	43.45	14.69	10	76					0.877**	0.778**	0.280**	0.669**
6 DIF	12.68	7.83	0	30						0.617**	0.01	0.461**
7 DDF	11.12	4.60	0	20							0.06	0.375**
8 EOT	9.17	3.94	0	18								−0.05
9 LSS	10.48	4.68	0	20								1

GAD Generalized Anxiety Disorder; PARQ Mother and PARQ Father acceptance/rejection- Higher scores indicate more acceptance and less rejection; TAS general level of alexithymia; DIF Difficulty identifying feelings; DDF Difficulty describing feelings; EOT Externally oriented-thinking; LSS Lack of subjective significance or importance of emotions.

*p < 0.05; **p < 0.01.

Both the relationship with the father and the mother has a significant negative relationship with alexithymia and its specific subdimensions. The higher the level of acceptance of both the mother and the father, the lower the level of alexithymia among adolescents. Interestingly, the strength of dependency in the relationship with the mother is only slightly higher than in the relationship with the father, suggesting an important role for fathers in the development of the child’s emotional competence. The result for the externally oriented thinking is noteworthy, as it is not significant. However, the dimension related to the lack of importance of emotions is significant. Similar to the other dimensions, this one also shows higher levels when parental acceptance is lower (Table 1).

Another important result from the perspective of the corroboration of the theoretical model is the significant relationship between parental rejection and GAD symptoms among adolescents. The strength of the acceptance/rejection relationship with the mother is greater, but the relationship between the relationship with the father and the symptoms of GAD is also statistically significant.

Finally, it is crucial to mention the strong relationship between GAD and the level of alexithymia and its dimensions outside of the externally oriented thinking. These results confirm that alexithymia, which is also significantly associated with parental rejection, may be an important factor in the development of anxiety disorders among adolescents (Paniccia et al., 2018). Lastly, it is worth noting that the relationship between the age of the participants and the other variables is insignificant, with the exception of the DDF dimension, which correlates with age at a positive, low level (Table 1).

Moving on to the confirmation of the theoretical model, the mediating role of alexithymia in the relationship between parental rejection (from both mother and father) and the symptoms of GAD is examined. The validation of the model was divided into two stages.

The first involves checking whether the overall level of alexithymia mediates the relationship between rejection by the mother or father and the symptoms of GAD, while the second focuses on identifying which dimension of alexithymia serves as the strongest mediator in each relationship.

**Figure 1:**
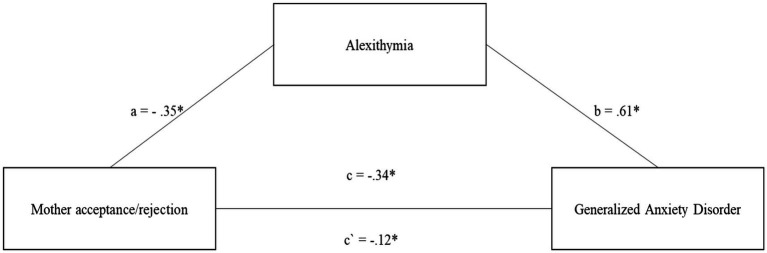
The mediating role of alexithymia in the relationship between Mother acceptance/rejection and the level of generalized anxiety disorder symptoms. All coefficients displayed are standardized estimates to ensure comparability across variables Mother acceptance/rejection—Higher scores indicate more acceptance and less rejection. c—The direct effect of the relationship between Mother acceptance/rejection and the level of generalized anxiety disorder symptoms. a—The relationship between Mother acceptance/rejection and alexithymia. b—The relationship between alexithymia and the level of generalized anxiety disorder symptoms. c`—The relationship between Mother acceptance/rejection and the level of generalized anxiety disorder symptoms after introducing alexithymia into the model. *p < 0.05; **p < 0.01.

The first model for the mediating role of alexithymia in the relationship between levels of rejection/acceptance in the relationship with the mother and the intensity of GAD symptoms is well fitted to the data *F* (2.220) = 87.47; *p* < 0.001 and explains 44.3% of the variability in GAD symptoms (Figure 1). The dynamic of acceptance/rejection in the relationship with the mother serves as a protective factor against the development of GAD (*c* = −0.34; *p* < 0.05). The same is true for the acceptance/rejection relationship with the mother with the level of alexithymia (*a* = −0.35; *p* < 0.05) (Figure 1).

On the other hand, the higher the level of alexithymia, the greater the risk of developing GAD (*b* = 0.61; *p* < 0.001). After incorporating alexithymia into the model, the strength of the relationship between acceptance/rejection in the relationship with the mother decreased but remained significant (*c*´ = −0.12; *p* < 0.05) (Figure 1). The mediation analysis can be considered partial and significant, which is confirmed by the results of the indirect and total effect *b* = 0.16, 95% CI [−0.22; −0.10]; *b* = 0.22, 95% CI [−0.28, −0.14].

**Figure 2:**
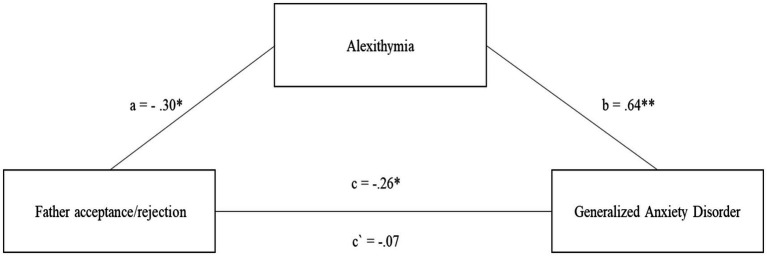
The mediating role of alexithymia in the relationship between Father acceptance/rejection and the level of generalized anxiety disorder symptoms. All coefficients displayed are standardized estimates to ensure comparability across variables Father acceptance/rejection—Higher scores indicate more acceptance and less rejection. c—The direct effect of the relationship between Father acceptance/rejection and the level of generalized anxiety disorder symptoms. a—The relationship between Father acceptance/rejection and alexithymia. b—The relationship between alexithymia and the level of generalized anxiety disorder symptoms. c`—The relationship between Father acceptance/rejection and the level of generalized anxiety disorder symptoms after introducing alexithymia into the model. *p < 0.05; **p < 0.01.

The second model, examining the mediating role of alexithymia in the relationship between rejection/acceptance in the relationship with the father and the severity of GAD symptoms, also fits the data well *F* (2.220) = 84.41; *p* < 0.001 and explains 43.4% of the variability in GAD symptoms (Figure 2).

The levels of acceptance/rejection in the relationship with the father acts as a protective factor in development of GAD (*c* = −0.26; *p* < 0.05) (Figure 2). The same is true for the relationship between acceptance/rejection in the relationship with the father and the level of alexithymia (*a* = −0.30; *p* < 0.05) (Figure 2). On the other hand, the higher the level of alexithymia, the greater the risk of developing GAD (*b* = 0.64; *p* < 0.001). After introducing alexithymia into the model, it turned out that the relationship between acceptance/rejection in the relationship with the Father declined to an insignificant level (*c*´ = −0.07; n.s.) (Figure 2). The mediation analysis can be considered full and significant, which is confirmed by the results of the indirect and total effect *b* = 0.11, 95% CI [−0.16; −0.06]; *b* = 0.19, 95% CI [−0.27, −0.12].

Comparing both models of mediation for the relationship with the Mother and the Father, it is important to highlight that acceptance/rejection in the relationship with the mother is a stronger protective factor in relation to alexithymia and GAD. However, the model for acceptance/rejection in the relationship with the father turned out to be a full mediation, while for the relationship with the mother it was only partial mediation.

After presenting mediation models that took into account the overall level of alexithymia, a number of models were validated to see which dimension of alexithymia would prove to be the strongest mediator. The importance of this analysis stems from the fact that the version of the instrument used for adolescents evaluates four subdimensions of alexithymia, in contrast to the three assessed by most standard versions. At this stage, four separate mediation models were tested to examine the relationship between maternal rejection and symptoms of generalized anxiety disorder (GAD), each model incorporating one of the alexithymia subdimensions (difficulty identifying feelings, difficulty describing feelings, externally oriented thinking, and Lack of subjective significance or importance of emotions) as a potential mediator.

At this stage, four mediation models were tested for the relationship between mother rejection and GAD, with the mediating role of alexithymia subdimensions. All models were found to be significant, respectively for DIF *F* (2,220) = 98.39, *p* < 0.001; 47%, DDF *F* (2,220) = 49.58, *p* < 0.001; 31.1%, LSS *F* (2,220) = 31.24, *p* < 0.001; 22.1%, and one model for EOT was also significant *F* (2,220) = 14.02, *p* < 0.001; 11.3%.

**Figure 3:**
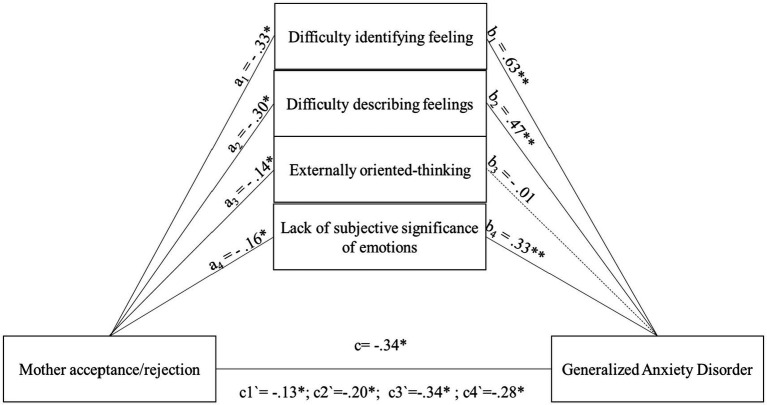
The mediating role of subscales of alexithymia in the relationship between Mother acceptance/rejection and the level of generalized anxiety disorder symptoms. All coefficients displayed are standardized estimates to ensure comparability across variables Mother acceptance/rejection—Higher scores indicate more acceptance and less rejection. c—The direct effect of the relationship between Mother acceptance/rejection and the level of generalized anxiety disorder symptoms. a1,b1—The mediating effect for the subdimension of alexithymia difficulty identifying feelings a2,b2—The mediating effect for the subdimension of alexithymia difficulty describing feelings a3,b3—The mediating effect for the subdimension of alexithymia externally oriented thinking a4,b4—The mediating effect for the subdimension of alexithymia lack of subjective significance of emotions. c1`–c4`—The relationship between Mother acceptance/rejection and the level of generalized anxiety disorder symptoms after introducing different alexithymia subscales into the model. *p < 0.05; **p < 0.01.

However, due to the absence of a statistically significant association between this dimension and generalized anxiety disorder (GAD), it cannot be regarded as theoretically meaningful. Nevertheless, for comparative purposes with alternative models, the associations involving the EOT dimension as a mediator are described and reported (Figure 3). Subsequently, the relationship between acceptance/rejection in the relationship with the mother and specific dimensions of alexithymia was examined, as illustrated in Figure 3. The results indicate significant associations for DIF (*a1* = −0.33; *p* < 0.05), DDF (*a2* = −0.30; *p* < 0.05), EOT (*a3* = −0.14; *p* < 0.05), and LSS (*a4* = −0.16; *p* < 0.05). As predicted, the strongest acceptance/rejection relationship with the mother was found to be for DIF and DDF. Then, the relationship between the dimensions of alexithymia and the level of GAD symptoms was examined. The strongest relationship was shown for the dimension DIF (*b1* = 0.60; *p* < 0.05). A slightly lower result was obtained for DDF (*b2* = 0.47; *p* < 0.05) and LSS (*b4* = 0.33; *p* < 0.05), and for EOT the association with the level of GAD symptoms was negligible EOT (*b3* = 0.01; n.s.) (Figure 3). After the introduction of mediators into the mediation models, the direct maternal acceptance/rejection relationship decreased across each alexithymia subdimension, reaching the following level: DIF *c´1* = −0.13; *p* < 0.05; DDF *c´2* = −0.20; *p* < 0.05; EOT *c´3* = −0.34; *p* < 0.05; LSS *c´4* = −0.28; *p* < 0.05 (Figure 3).

Among the reported associations, as previously noted, the mediation effect of the EOT dimension cannot be considered significant. However, for the remaining three dimensions, partial mediation was observed, as confirmed by the significant indirect and total effects for DIF *b* = 0.15, 95% CI [−0.22; −0.09], *b* = 0.21, 95% CI [−0.29, −0.13]; DDF *b* = 0.10, 95% CI [−0.16, −0.05]; *b* = 0.14, 95% CI [−0.21, −0.07]; LSS *b* = 0.04, 95% CI [−0.07, −0.01]; *b* = 0.05, 95% CI [−0.10, −0.01].

**Figure 4:**
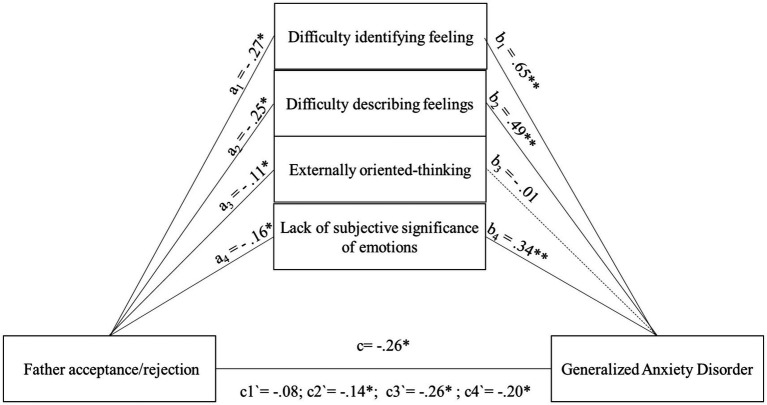
The mediating role of subscales of alexithymia in the relationship between Father acceptance/rejection and the level of generalized anxiety disorder symptoms. All coefficients displayed are standardized estimates to ensure comparability across variables Father acceptance/rejection—Higher scores indicate more acceptance and less rejection. c—The direct effect of the relationship between Father acceptance/rejection and the level of generalized anxiety disorder symptoms. a1,b1—The mediating effect for the subdimension of alexithymia difficulty identifying feelings a2,b2—The mediating effect for the subdimension of alexithymia difficulty describing feelings a3,b3—The mediating effect for the subdimension of alexithymia externally oriented thinking a4,b4—The mediating effect for the subdimension of alexithymia lack of subjective significance of emotions. c1`–c4`—The relationship between Father acceptance/rejection and the level of generalized anxiety disorder symptoms after introducing different alexithymia subscales into the model. *p < 0.05; **p < 0.01.

As in the case of acceptance/rejection in the relationship with the mother, four models were also tested in the relationship with the father, three of which were found to be relevant, respectively, for DIF *F* (2.220) = 94.96; *p* < 0.001; 46.3%, DDF *F* (2.220) = 45.47; *p* < 0.001; 29%; LSS *F* (2.220) = 24.56; *p* < 0.001; 18.3%. The model for the EOT dimension is also statistically significant, F (2.220) = 7.96; *p* < 0.001, explaining 6.8% of the variance. However, due to the absence of a significant relationship between EOT and GAD, it cannot be considered theoretically meaningful. Nonetheless, as in the case of the Mother Acceptance/Rejection model, it will be discussed for comparative purposes (Figure 4). The next step was to examine the relationship between levels of acceptance/rejection in the relationship with the father and specific dimensions of alexithymia, which are outlined as follows: DIF (*a1* = −0.27; *p* < 0.05). DDF (*a2* = −0.25; *p* < 0.05). EOT (*a3* = −0.11; *p* < 0.05) and LSS (*a4* = −0.16; *p* < 0.05) (Figure 4). As predicted, the strongest acceptance/rejection relationship with the father turned out to be for DIF and DDF. Then, the relationship between the dimensions of alexithymia and the level of GAD symptoms was examined. The strongest relationship was shown for the dimension DIF (*b1* = 0.65; *p* < 0.05). A slightly lower result was obtained for DDF (*b2* = 0.49; *p* < 0.05) and LSS (*b4* = 0.34; *p* < 0.05), and for EOT the association with the level of GAD symptoms was negligible EOT (*b3* = 0.01; n.s.) (Figure 4). After the introduction of mediators into the mediation model, the direct relationship for father acceptance/rejection decreased for each alexithymia subdimension to the following level: DIF *c´1* = −0.08; n.s. DDF *c´2* = −0.14, *p* < 0.05; EOT *c´3* = −0.26, *p* < 0.05; LSS *c´4* = −0.20, *p* < 0.05 (Figure 4). Among all the relationships discussed, one case of full mediation was observed for the DIF dimension, along with two partial mediations for the DDF and LSS dimensions, and not significant mediation effect for the EOT dimension. These conclusions are supported by the significant results of the indirect and total effects for DIF *b* = 0.10, 95% CI [−0.15; −0.05]; *b* = 0.18, 95% CI [−0.26; −0.10]; DDF *b* = 0.07, 95% CI [−0.11; −0.03]; *b* = 0.12, 95% CI [−0.19; −0.05]. LSS *b* = 0.03, 95% CI [−0.05; −0.01]; *b* = 0.06, 95% CI [−0.10; −0.01].

## Discussion

The aim of this study was to investigate the relationship between levels of maternal and paternal rejection and GAD in early adolescents, examining the possible mediating role of alexithymia. To our knowledge, this is the first study to examine the relationship between parental rejection and GAD in a sample of early adolescents and attempts to extend knowledge of a possible mediating factor, namely alexithymia. Early adolescence is a critical developmental period, and individuals at this age appear to be at risk of developing anxiety symptoms, particularly GAD. This anxiety syndrome is one of the main reasons for parents to seek psychological intervention for their children. Given the severe impact of GAD on adolescents’ social and academic functioning, it is important to understand what risk factors and mechanisms may play a role.

Our results show a relationship between parental rejection and the risk of GAD in early adolescents. Specifically, when adolescents report a relationship characterized by low levels of acceptance and high levels of rejection, they tend to report more GAD symptoms. These data are consistent with previous literature identifying parental rejection as a possible risk factor for adolescent psychological adjustment (Khaleque and Rohner, 2012) and are in line with previous studies that have found an association between parental rejection and GAD (Hale III et al., 2006; Knappe et al., 2022). In this sense, it is possible that the experience of parental rejection influences the mental representation that individuals develop of themselves as unlovable and of others as subjects prone to abandonment and criticism, fueling the concerns that increase the risk of developing GAD.

Furthermore, our data show that both maternal and paternal rejection directly and indirectly predicted GAD symptoms. This finding is interesting because it confirms the contribution that both parents could make to the occurrence of GAD. The Acceptance/Rejection theory has received solid empirical support for the role of both parents in predicting psychological adjustment in adolescents and young adults (Miranda et al., 2016; Khaleque and Rohner, 2012), while questions remain about the possible association between maternal and paternal rejection and specific developmental outcomes in children, adolescents and adults (Mendo-Lázaro et al., 2019). While some previous findings tend to distinguish the effects of maternal and paternal parenting on the onset of anxiety symptoms (Beato et al., 2016), our study shows how parental rejection significantly predicts GAD symptoms in both the relationship with the mother and the father. Our data are consistent with previous literature showing an association between internalizing symptoms and parental rejection, both maternal and paternal (Giotsa et al., 2018; Giaouzi and Giovazolias, 2015). The only available evidence on the relationship between parental rejection and specifically GAD symptoms in adolescence has examined only the overall level of perceived parental rejection, and therefore further future research is needed to compare our data with other studies. Furthermore, our study shows not only that both paternal and maternal rejection are associated with GAD, but also that the relationship between GAD and parental rejection is stronger for maternal rejection. Some cultural factors may help to understand this. Namely, from a cultural perspective, mothers are entrusted with the task of caring for their children and tend to spend much more time with their children than fathers. Thus, it is possible that children and adolescents are exposed to more criticism and rejecting behavior in the maternal relationship, which could reinforce the association between maternal rejection and GAD compared to paternal rejection. However, there are other explanations. For example, it is possible that mothers, precisely because they are culturally primarily responsible for raising their children, are more likely to resort to disciplinary measures than fathers, which could reinforce adolescents’ perceptions of maternal rejection. In addition, mothers tend to be more at risk of parental burnout (Sorkkila and Aunola, 2020; Scigala et al., 2024) and in this situation it is possible that they are more likely to engage in negative parenting behaviors (Scigala et al., 2024), which could have a greater impact on adolescents’ perceptions of maternal rejection. It is therefore possible that children and adolescents are more intensely confronted with maternal rejection. This may shape the way in which the individual represents themselves and the relationship with others, fueling GAD symptoms. On the other hand, we should point out that the gendered, socially and culturally determined roles that bind mothers to childcare might nurture greater expectations of care, protection and affection from maternal figures than from paternal figures. In this sense, a negative relationship with the mother could reinforce the adolescents’ perception of maternal rejection. However, further research is needed to understand this finding.

Although our data show a stronger association between GAD symptoms and maternal rejection, we should not forget that our data indicate a strong association between GAD symptoms and paternal rejection in adolescents. This finding is important because it also attributes a possible role to the relationship with the father in the onset of GAD symptoms in adolescents. Indeed, the literature has largely ignored the role of the paternal relationship in the development or maintenance of anxiety symptoms in children and adolescents, attributing greater importance to the maternal figure (Bögels and Phares, 2008). This finding prompts us not only to further explore the unique contribution of the paternal figure in relation to GAD symptoms, but also to reflect on the paternal role in a cultural context such as the Italian one, where the maternal figure still tends to occupy the role of primary caregiver, suggesting a greater maternal responsibility in the processes of care, education and growth of children and adolescents.

Our study also shows a significant association between parental rejection (both maternal and paternal) and alexithymic symptoms. This finding is consistent with the literature that attributes an important role to the relationship with caregivers in shaping an individual’s emotional functioning (Cooke et al., 2019). The research data show that both adolescents (Lukas et al., 2022) and adults (Huang et al., 2022) with alexithymia tend to perceive the relationship with their parents as rather negative, characterized by low levels of affection and high levels of criticism and rejection. Parental rejection is seen as a form of emotional abuse that contrasts with the emotional needs for protection, affection and closeness that children express in the primary relationship with the caregiver. In such a relational context, the child is likely to develop an insecure attachment to the caregiver and lack a relationship that supports the child in developing appropriate emotional regulation skills. It is therefore possible that the child growing up in a similar relational context with the caregiver will increasingly use maladaptive emotion regulation strategies, such as suppression of emotions (van Strien et al., 2019). In this way, the child would have difficulty developing skills related to identifying and verbalizing their emotions, increasing the risk of developing alexithymic traits.

In addition, our study revealed the possible mediating role of alexithymia in the relationship between parental rejection and GAD. Specifically, the analyses showed that the three sub-dimensions of alexithymia, relating to “difficulty identifying feelings” (DIF), “difficulty describing feelings” (DDF), and “lack of subjective significance or importance of emotions” (LSS), were significant in mediating the relationship between maternal and paternal parental rejection and GAD, whereas the “externally-oriented thinking” (EOT) dimension was not significant. These data are in line with the research findings of Paniccia et al. (2018), who found in a sample of Italian adolescents that adolescents with GAD had higher scores specifically in the two sub-dimensions of alexithymia, namely “difficulty identifying feelings” (DIF) and “difficulty describing feelings” (DDF), compared to their peers without alexithymia. Similarly, in a comparison of adults with and without a GAD diagnosis, Marchesi et al. (2005) found no significant differences in the alexithymia sub-dimension of “externally-oriented thinking” (EOT), but found that the significant increase in alexithymia in GAD patients depended on the sub-dimension of “difficulty identifying feelings” (DIF). Overall, these data seem to suggest that it may be the affective components of alexithymia that play a role in the onset and maintenance of anxiety symptoms, particularly in GAD. Other evidence seems to point in the same direction. For example, Işıl (2023) recently found that the two sub-dimensions of alexithymia, “difficulty identifying feelings” (DIF) and “difficulty describing feelings” (DDF), are significant in mediating the relationship between remembered maternal rejection and somatic complaints in adults, whereas the subdimension “externally-oriented thinking” (EOT) is not (Işıl, 2023). In addition, our study identified the possible mediating role of the alexithymia subdimension “lack of subjective significance or importance of emotions” (LSS). There are no other studies that have examined this subdimension with which to compare our data, but an explanation could be offered. Indeed, individuals who have experienced a relationship with their parents characterized by rejection and criticism might be more prone to develop avoidant attachment, which is characterized by a marked tendency of the individual to deny their needs for emotional closeness, avoid emotional involvement and intimacy (Fabris et al., 2018). It is therefore likely that these individuals have learned to underestimate the importance of emotions and that this attitude helps them to reduce the occasions for emotional solicitation in interpersonal relationships. Individuals with avoidant attachment tend to use more emotion suppression strategies, and there is evidence that this is the most prevalent form of insecure attachment in the population of individuals diagnosed with GAD (Newman et al., 2016). However, further research is needed.

Overall, our data suggest that parental rejection (both maternal and paternal) is directly and indirectly through alexithymia, associated with the risk of GAD in early adolescents. In particular, our data seem to support the hypothesis that adolescents who experience a relationship with caregivers characterized by rejection and criticism tend to have a higher risk of reporting GAD symptoms, and that certain sub-dimensions of alexithymia (DIF, DDF, LSS) may partially explain this association. It is possible that parental rejection undermines the development of emotion regulation skills and causes the affected individual to resort more frequently to maladaptive emotion regulation strategies, such as emotional suppression, increasing the risk of developing alexithymic symptoms. In this sense, alexithymia, and in particular the difficulty in recognizing and verbalizing one’s own emotions, can make it difficult for the affected person to cope with and regulate negative and painful feelings. In order to avoid experiencing distressing negative emotions and the associated autonomic arousal, GAD individuals tend to use the abstract, verbal–linguistic process of worry (Cassidy et al., 2009). In practice, adolescents with GAD may attempt to defend or distance themselves from negative feelings by shifting their attention from negative emotions toward abstracted, conceptual thoughts (i.e., worry) (Mennin et al., 2002, 2005). Ultimately, worry can be seen as an extreme and maladaptive attempt to down-regulate anxiety triggered by uncertain, unpredictable or uncontrollable situations that trigger particularly strong negative emotions (Borkovec et al., 2004).

In summary, our study found that parental rejection is associated with increased symptoms of Generalized Anxiety Disorder (GAD) in early adolescents, with alexithymia as a potential mediator. Adolescents experiencing low affection and high rejection from parents are at greater risk of GAD due to difficulties in developing emotional skills, which hinders their ability to identify and verbalize emotions. This can lead to challenges in regulating negative emotional states, resulting in increased reliance on worry as a coping mechanism, further elevating the risk of GAD.

### Limits and future directions

Although our data contribute to expanding the literature on possible factors involved in the development or maintenance of GAD in early adolescence, these data must be read with serious consideration of the methodological limitations of our study. Namely, the data come from a cross-sectional study. Therefore, it is not possible for us to express ourselves in terms of linear causality. In this sense, it is not possible to determine, for example, whether previous experiences of parental rejection cause the increase in GAD symptoms, or whether GAD symptoms influence the way adolescents perceive or interpret the quality of relationships with their parents, or whether adolescents with GAD symptoms engage in behaviors that attract more negative parental behaviors, such as parental rejection. In this direction, future studies could replicate the investigation in a longitudinal research design. Notably, the current study design also did not allow for the testing of alternative models regarding the directionality of the relationships between parental rejection, alexithymia, and GAD symptoms. As mentioned above, it is plausible that GAD symptoms or alexithymia influence perceptions of parental rejection and not vice versa. Future research should utilize longitudinal studies or experimental manipulations to decipher these potential causal pathways. Furthermore, the generalizability of our study is severely limited by the sample size, the nationality of the respondents, and the fact that our study is a convenience sample of the general population. Therefore, the sample is not representative of the population of Italian adolescents. Future studies could use larger, representative samples and, if possible, propose cross-cultural surveys to test the generalizability of our data to other cultural contexts. In addition, our sample is not a clinical sample and is limited to the identification of symptoms indicative of GAD. Future studies could therefore include adolescents with a formal diagnosis of GAD. Finally, we only used self-report instruments. This is a limitation because factors such as text comprehension, social desirability, and the subjects’ emotional state may have influenced the adolescents’ responses. In addition, the interpretation of parental rejection is limited by the fact that it relies on self-report measures, which may reflect perceived rather than actual parental behavior and whose accuracy may be compromised due to retrospective measurement. Future studies could include observational data from parents, peer reports or clinical assessments to obtain a more comprehensive view. Therefore, future studies could consider not only clinical samples of adolescents with GAD, but also include other instrument types or observers to explore the relationship between the variables examined here.

### Practical implications

Our study suggests some possible practical implications related to adolescents’ mental wellbeing. Firstly, it is important to make parents (both mothers and fathers) aware of the impact that parenting characterized by coldness, rejection, criticism and hostility can have on the emotional development and adjustment of their children during puberty. It is important to try to promote more positive relationships characterized by affection, closeness and support in the parent–child relationship, and especially to intervene in risk situations. In this direction, mental health professionals could work on the recognition and regulation of emotions in adolescents exposed to situations in which the parental relationship is characterized by parental rejection. In addition, for adolescents with GAD symptoms, they could carefully consider the co-existence of alexithymic symptoms and assess the possible contribution of the parental relationship, especially if they have experienced parental rejection. In this case, clinical intervention with adolescents with GAD might emphasize processing parental rejection and promote greater awareness of their own emotional and relational functioning to help them reduce alexithymic symptoms. On the other hand, it might be useful to try to engage adolescents in training aimed at recognizing, identifying, and managing emotions as part of the treatment pathway for anxiety symptoms.

## Data Availability

The raw data supporting the conclusions of this article will be made available by the authors without undue reservation.
